# Devastating salt‐wasting crisis in a four‐month‐old male child with congenital adrenal hyperplasia, highlighting the essence of neonatal screening

**DOI:** 10.1002/ccr3.6010

**Published:** 2022-07-11

**Authors:** Nagaspurthy Anugu Reddy, Sucheta Sharma, Mainak Das, Ashutosh Kapoor, Upasana Maskey

**Affiliations:** ^1^ Suraksha Women and Children Hospital Hyderabad India; ^2^ Punjab Institute of Medical Sciences Jalandhar India; ^3^ Nilratan Sircar Medical College Kolkata India; ^4^ NHS Redditch UK; ^5^ Everest Hospital Kathmandu Nepal

**Keywords:** CAH, congenital adrenal hyperplasia, neonatal screening

## Abstract

Congenital adrenal hyperplasia (CAH) is a rare condition usually referred to as a group of genetic disorders resulting due to a deficiency of steroid enzymes required by adrenal glands to produce cortisol and mineralocorticoid hormones. It has an autosomal recessive mode of inheritance and is further categorized into two types—Classic and Non‐Classic. Non‐Classic CAH is a more common milder form that presents late after puberty. Classic CAH, although more severe, is rare and detected at birth and is associated with the life‐threatening adrenal crisis in both sexes and virilization of the external genitalia in females (46, XX) patients, whereas in males, no overt abnormality of the external genitalia is present. We present a case of a four‐month‐old male child with the classic form of CAH who was brought with complaints of loose stools, projectile non bilious vomiting, decreased urine output, and failure to feed for 3 days. The child had a clinical presentation of salt wasting with hypoglycemia and hyperpigmentation of his genitalia. The USG findings revealed increased anteroposterior diameter of renal pelvis indicative of a growth in the suprarenal area. 17‐hydroxyprogesterone (17‐OHP) was found to be elevated confirming the diagnosis. He was treated with hydrocortisone with gradual improvement in his glucose and electrolytes. The patient was discharged home on replacement therapy consisting of oral prednisolone and fludrocortisone acetate and followed up as outpatient with significant improvement in the clinical findings. The fact that the child was not screened for CAH at birth led to the critical consequences of the disease in this case. To prevent life‐threatening adrenal crisis and help perform appropriate sex assignments for affected female patients, newborn screening (NBS) programs for the classical form of CAH should be made mandatory even in low‐ and middle‐income countries.

## Graphical abstract

Congenital adrenal hyperplasia although rare can present with a devastating salt‐wasting crisis, virilization of females, and failure to thrive. Early diagnosis through screening at birth can yield better outcomes. This case highlights the need for mandatory screening in low‐ and middle‐income countries.

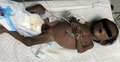

## 1 INTRODUCTION

Congenital adrenal hyperplasia (CAH) is a group of inherited disorders caused by genetic defects that hinder the production of adrenal hormones, cortisol, and aldosterone, either completely or at their normal rate. It may be caused by a deficiency of one of the five enzymes responsible for cortisol synthesis. These enzymes include 21α‐hydroxylase, 11β‐hydroxylase and 17α‐hydroxylase/17,20‐lyase, cholesterol 20,22 desmolase, and 3β‐hydroxysteroid dehydrogenase.^1^ Adrenal steroidogenesis occurs by a series of steps facilitated by the zone‐specific enzyme expression and different types of CAH interrupt this process at distinct branch points. Furthermore, there is a substitute pathway for the endogenous synthesis of androgens, which also plays a significant role in the causation of CAH.^2^, ^3^ CAH is further categorized into two types—Classic and Non‐Classic. Classic CAH, although a more severe form, is rare and is usually detected at birth and is associated with the life‐threatening adrenal crisis in both sexes and virilization of the external genitalia in 46, XX patients. Non‐Classic CAH is a more common form that has a milder phenotype in which clinical problems are not obvious during the neonatal period or childhood and generally develops during adolescence or adulthood.^4^, ^5^ In Classic CAH, the findings usually visible at birth include ambiguous genitalia in females due to excess male androgens, whereas in males, no overt abnormality of the external genitalia is present. In 95% of the cases of CAH, the identified deficiency is of 21α‐hydroxylase enzyme.^6^ This leads to impairment of cortisol and aldosterone production with excess production of androgens that leads to the aforementioned findings. We present a case of a four‐month‐old male child who presented with Classic CAH with signs of dehydration, malnutrition, and failure to thrive.

## 2 CASE PRESENTATION

A four‐month‐old male child was brought to the emergency department by the parents with complaints of loose stools, projectile non‐bilious vomiting, decreased urine output, and failure to feed for 3 days.

Birth History—The child was born at 37 weeks of gestation by assisted vaginal delivery (vacuum) with a birth weight of 2.5 kg. A single dose of betamethasone was given 9 h before the birth of the child. He had cried immediately after birth and the APGAR score was five at 1 min and eight at 5 min. Injection Vit K with Vit D3 oral drops was given after birth. Routine immunization as per the National Healthcare System (NHS) was done and breastfeeding was initiated in the hospital, and he was exclusively breastfed to date. He was the first child of a non‐consanguineous couple. There was no family history of any chronic or genetic diseases.

**FIGURE 1:**
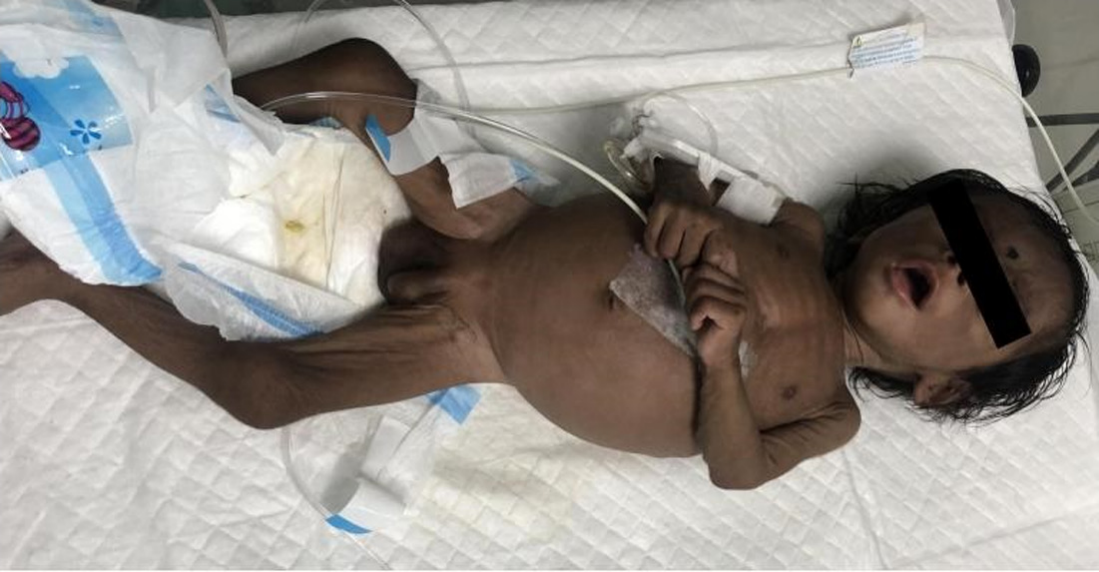
Male genitalia in a four‐month‐old infant showing hyperpigmentation accompanied by features severe malnourishment and muscle wasting with clinical signs in keeping with life‐threatening dehydration

During the present visit at 4 months of age, the child looked dehydrated with sunken eyes and dry oral mucosa. On thorough clinical examination of the child, the weight was 3.5 kg and body length was 60 cm. Mild‐to‐moderate fever (temp: 100 °F) and irritability were noted. The blood pressure was 70/36 mmHg, heart rate was 112 bpm, resp rate was 25/min, SpO2 was 92% at room air, and 97% on two liters of oxygen. Examination of the respiratory system, cardiovascular, and abdomen were within normal limits. The male genitalia were correctly identified, hyperpigmentation of the overlying skin with clinical signs consistent with severe muscle wasting and life‐threatening dehydration was observed as shown in Figure 1. With the current body weight of 3.57 kg (i.e., below 10th percentile), the child was labeled under failure to thrive. The patient was admitted to the pediatric inpatient unit with a provisional diagnosis of acute gastroenteritis with dehydration and hypoglycemia for observation and further management.

Investigations showed deranged electrolytes with serum sodium: 119 mmol/L, serum potassium: 1.7 mmol/L, and serum creatinine: 61.89 μmol/L. Random blood sugar was 0.89 mmol/L. The hyponatremia and hypokalemia were assumed to be caused by dehydration owing to diarrhea and vomiting from the past 3 days. His initial blood gas analysis showed: pH‐ 7.54 (7.35–7.45), pCO2 3.99 kPa (4.27–6), HCO_3_ 27.3 mmol/L (22–28) suggestive of respiratory alkalosis. Normal levels of magnesium (0.74 mmol/L) and ionized calcium (1.11 mmol/L) successfully ruled out the possibility for Bartter and Gittelman syndrome. The laboratory results are outlined in Table 1. The stool for reducing substances was found to be negative. C‐peptide was done to rule out insulinoma which was 0.15 ng/mL. Liquid chromatography—tandem mass spectrometry (LC–MS/MS) was done to rule out any inborn metabolic errors, which reported to have no obvious organic aciduria and no evidence of elevated metabolites.

**TABLE 1:** Laboratory findings (Pre‐Intervention)

Test	Value	Normal range	Test	Value	Normal range
Hematology			Arterial blood gas		
WBC	13.8 10^9^cells/L	(3.5–9.1)	pH	7.54	(7.35–7.45)
Hb	5.65 mmol/L	(7.01–9.43)	pCO_2_	3.99 kPa	(4.27–6)
Hct	28%	(33.4–44.9)	HCO_3_	27.3 mmol/L	(22–28)
Plt	490 10^9^cells/L	(150–450)			
Biochemistry			Endocrine		
S.Cre	61.89 μmol/L	(35.37–123.79)	17‐OHP	2.4 nmol/L	(0.06–0.27)
Na	119 mmol/L	(135–150)	Cortisol (8 am)	60.7 nmol/L	(165.5–634.5)
K	1.7 mmol/L	(3.5–5.5)	Urine		
Cl	98 mmol/L	(94–110)	Na	36 mmol/L	(40–220)
Ca (Ionized)	1.11 mmol/L	(1.10–1.35)	K	25 mmol/L	(20–125)
Mg	0.74 mmol/L	(0.62–0.82)	Cl	72 mmol/L	(100–250)
T‐bil	7.01 μmol/L	(3.42–18.81)			
AST	0.93 μKat/L	(0.17–0.66)			
ALT	0.43 μKat/L	(0.08–0.75)			
Glu (RBS)	0.89 mmol/L	(2.78–6.05)			
CRP	101,000 μg/L	<6000			

**FIGURE 2:**
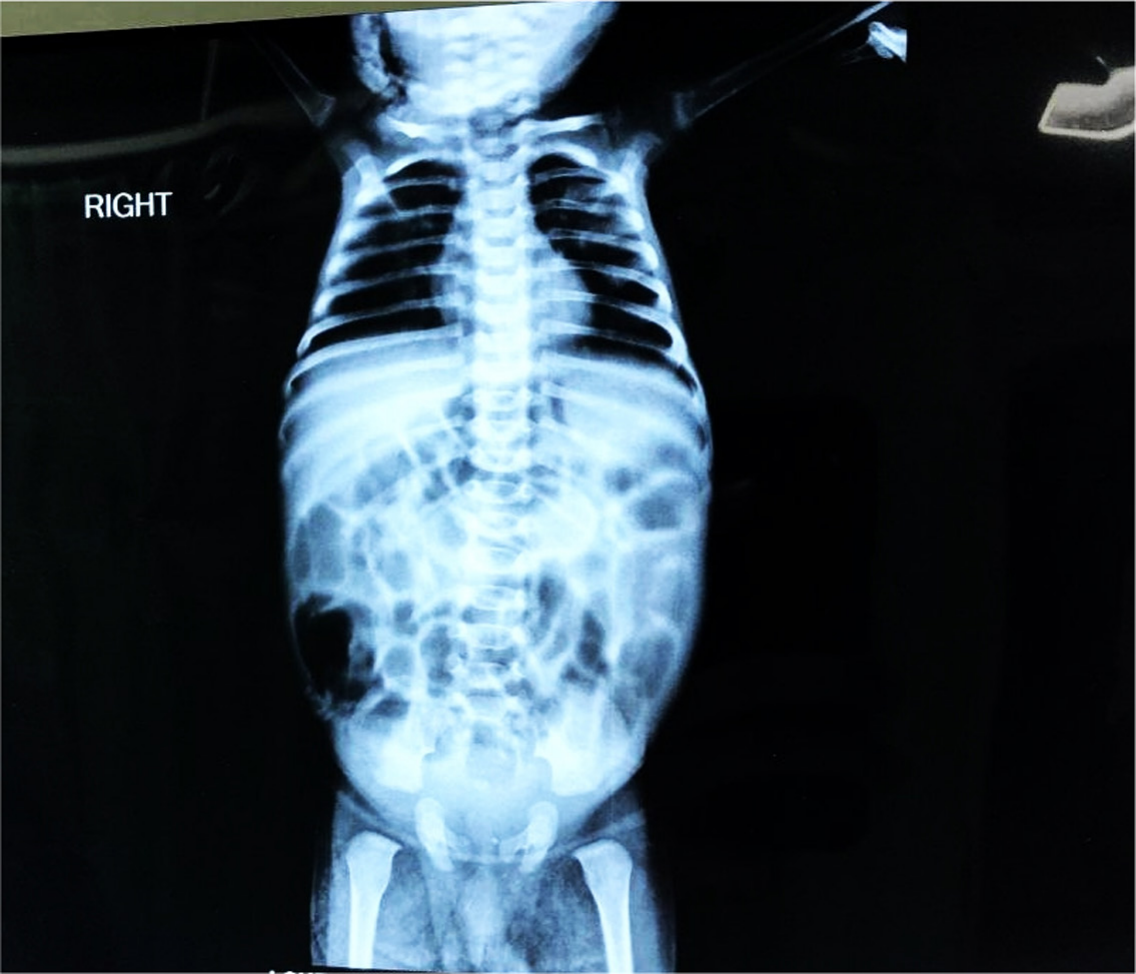
X‐ray erect abdomen showing normal findings

**FIGURE 3:**
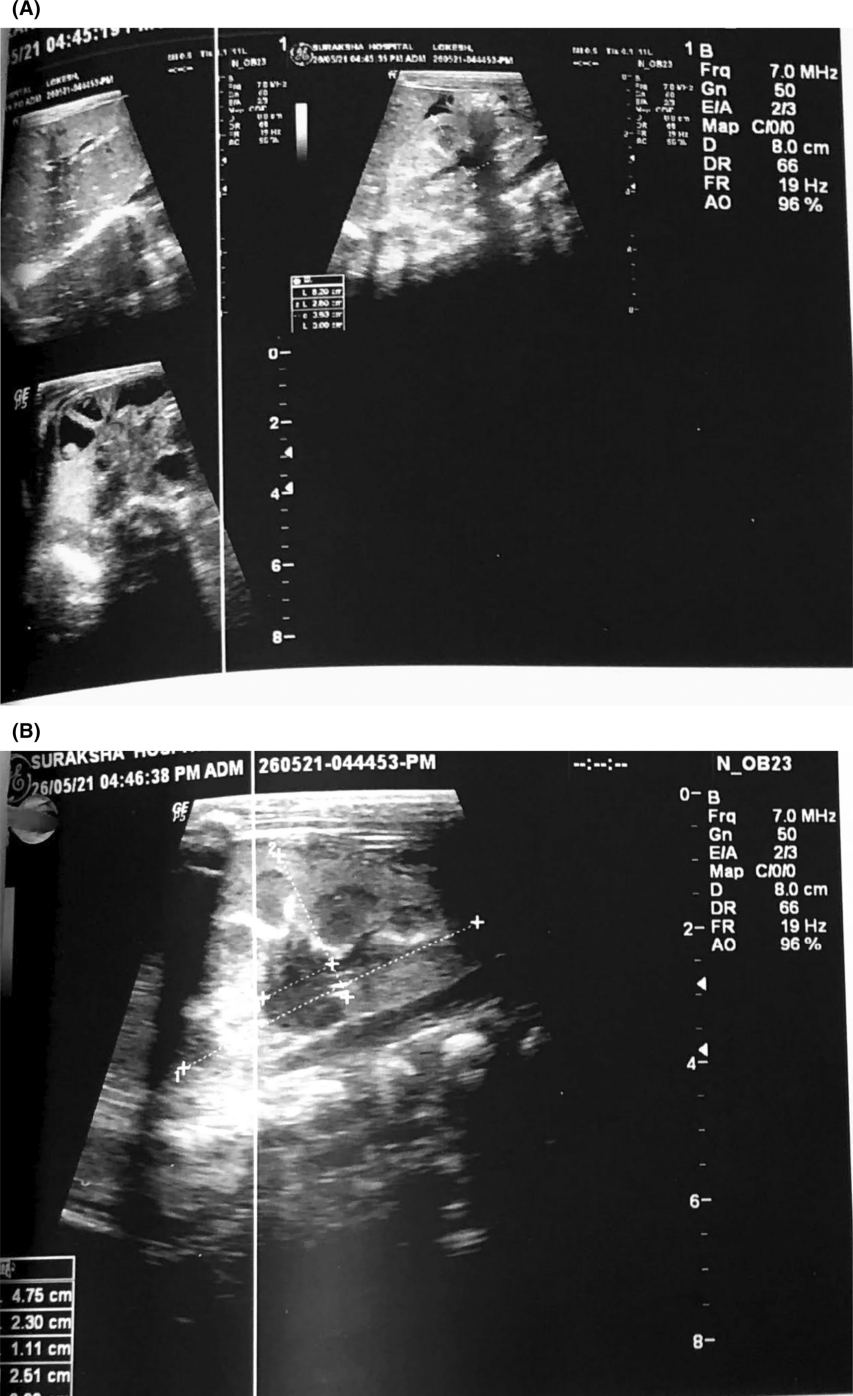
(A) USG showing bilateral increased renal echotexture. (B) Ultrasound showing a prominent left renal pelvicalyceal system with an 11 mm AP diameter of the renal pelvis

X‐ray erect abdomen was normal as shown in Figure 2. ECG was also found to be normal. Ultrasonography of the abdomen and pelvis (Figure 3A,B) showed bilateral increased renal echotexture probably due to dehydration and prominent left renal pelvicalyceal system with an anteroposterior diameter of renal pelvis 11 mm. The patient was given supportive measures and started on antibiotics to cover possible sepsis but the work‐up for sepsis later came back negative. The electrolyte levels on Day two showed Na‐119 mmoL/L, K‐6.0 mmol/L. The morning sample for Cortisol drawn at 8 am on Day two showed decreased levels of the hormone (60.7 nmol/L). Patient was started on hydrocortisone 15 mg IV eighth hourly with hourly monitoring of vitals. The blood was drawn and sent for 17‐hydroxyprogesterone (17‐OHP) for suspicion of CAH which was found to be elevated 2.4 nmol/L (normal: 0.06–0.27) indicative of the diagnosis. His glucose and electrolytes were monitored daily while in the hospital, which gradually improved by the seventh day of hospitalization. Table 2 depicts the electrolytes and blood glucose levels measured on 7th day of hospitalization, that is, after the replacement therapy was begun. Further investigations including genetic testing was refused by the parents of the child owing to financial reasons despite adequate counseling by the hospital. Gradual improvement in the clinical signs and symptoms was observed early after starting the replacement therapy. Given the potential concerns surrounding compliance and once daily dosing regimen of Prednisone in comparison to hydrocortisone, the decision was made to discharge the patient on take home prednisone with fludrocortisone acetate. Also, considering the socio‐economic status of the family, the market price difference between hydrocortisone and prednisone played a role in the assistive decision making. He was followed up after 7 days in the outpatient department. His weight had improved, electrolytes were normal, and medications were adjusted according to his weight.

**TABLE 2:** Laboratory findings (post‐intervention)

Test	Value	Normal Range
Biochemistry		
Na	135 mmol/L	(135–150)
K	5.5 mmol/L	(3.5–5.5)
Cl	103 mmol/L	(94–110)
Glu (RBS)	2.3 mmol/L	(2.78–6.05)

Abbreviations: 17‐OHP, 17‐hydroxyprogesterone; ALT, alanine aminotransferase; AST, aspartate aminotransferase; Ca, calcium; Cl, chloride; CRP, c‐reactive protein; Glu (RBS), glucose (random blood sugar); Hb, hemoglobin; Hct, hematocrit; K, potassium; Mg, magnesium; Na, sodium; Plt, platelet; S.Cre, serum creatinine; T‐bil, total bilirubin; WBC, white blood cell.

## 3 DISCUSSION

Classic congenital adrenal hyperplasia (CAH) is generally caused by mutations in the CYP21A2 gene (resulting in 21α‐hydroxylase deficiency) and has a prevalence of 1:15,000.^7^ The mode of inheritance is mainly autosomal recessive.^8^ The patients deficient in 21α‐hydroxylase present with signs of cortisol deficiency along with aldosterone deficiency in life‐threatening situations. Insufficiency of adrenal hormones disturbs the balance of the hypothalamus‐pituitary–adrenal (HPA) axis and impairs the negative feedback system leading to an increase in ACTH secretion from the pituitary gland. The stimulant effect of ACTH on the steroidogenesis pathway, in turn, leads to hyperplasia of both the adrenal glands with increased production of androgens. The intensity of clinical manifestations depends on the site and severity of the defect in the biosynthesis pathway.^8^ In this variety, there is an accumulation of 17‐OH‐progesterone in the serum, which is further redirected to form adrenal androgens.^8^ In about one‐third of the cases, this defect is severe and presents at birth with features of glucocorticoid and mineralocorticoid deficiency and elevated androgens. The remaining two‐third of the cases have adequate secretion of mineralocorticoids but there may be features of cortisol insufficiency and/or ACTH and androgen excess. Occasionally mild enzyme defects do not manifest until early adult life, whereas females may present with amenorrhea and/or hirsutism, mimicking clinical signs of PCOS^9^ which is referred to as late‐onset CAH^8^.

With the increasing concern on inherited defects, screening for CAH has been a part of the routine neonatal check‐up in many parts of the world. In Sweden and Norway, the test is performed by measuring 17 hydroxyprogesterone (17‐OHP) in blood using a filter paper no earlier than 48 hours after the birth. The other screening methods are radio‐immunoassays employed in the US, enzyme‐linked immunosorbent assay in Japan, and time‐resolved Fluro‐immunoassay in Europe. Preterm newborns have a higher 17‐OHP concentration in serum than babies born at term. Thus, cut‐off levels are based on gestational age in Japan and Europe, and birth weight in the US.^10^ Screening aims to determine the sex, improve the outcomes, and prevent neonatal deaths.^11^ During the prenatal period, methods such as amniocentesis and chorionic villous sampling (CVS) can be utilized to screen the fetus for genetic defects. The lack of adequate screening in this part of the world led to the severe clinical presentation in this child with typical signs of salt loss and hypoglycemia. It has been shown that these findings can have a negative influence on the child's cognitive performance. The long‐term consequences of the salt‐wasting crisis at birth are hard to predict, but early diagnosis and avoiding hypocortisolism in the neonatal period has proved to improve prognosis and lead to a favorable cognitive development as well.^7^

The management of this disorder is a constant balancing act. Although the prenatal administration of synthetic glucocorticoid (dexamethasone) to pregnant females with a previous history of a child with CAH has been shown to ameliorate virilization of external genitalia in the affected female fetus, uncertainties exist in terms of long‐term efficacy and safety profile of this measure or its use in male fetuses. Adding to the concern is the fact that the dose of dexamethasone that the fetus is exposed to is estimated to be 60 times the normal fetal cortisol level.^12^ The management involves the lifelong substitution of cortisol but due to the inability to match the exact circadian rhythm of the hormones, it carries a risk of both under and over‐treatment. Our patient improved significantly with the appropriate dose and treatment with normalization of the electrolytes and adequate weight gain. But a careful approach was warranted as the receptors for both glucocorticoids and mineralocorticoids are overly expressed in parts of the brain such as the hippocampus, amygdala, and prefrontal cortex and excess replacement may disturb the cognitive and affective functions in patients receiving overtreatment.^7^ Ideally, there should be a balance between the substitution and suppression of glucocorticoids and mineralocorticoids. Needless to say, patients with Classic CAH on replacement therapy require added care and monitoring especially during the critical stages of any illness. Administration of stress doses of hydrocortisone acutely during sickness undoubtedly plays a very crucial role but may still be unable to control hypoglycemia. Although our patient showed significant improvement in the glucose levels with the treatment, as per the guidelines, additional glucose supplementation is essential in preventing a hypoglycemic crisis, especially in the pediatric population.^13^

The potentially fatal presentation of the patient at the time of arrival to the hospital could have been prevented, had the child been screened for the disorder at birth. This case, therefore, highlights the significance of neonatal screening for CAH at birth for better outcomes in patients. The newborn screening for CAH has already been a routine part of the neonatal screening protocol for most countries^12^ and calls for an urgent need in low and middle‐income countries as well.

## 4 CONCLUSION

Classic congenital adrenal hyperplasia, although rare, can present with a devastating picture of hypoglycemia and salt‐wasting crisis as seen in this case. Although female patients present with signs of virilization, this male child presented with hyperpigmentation of his genitalia with additional concerns of failure to thrive. There is an increased risk for cognitive impairment as a consequence of the presentation seen with this disorder and thus timely diagnosis and management are crucial. This case highlights the need for neonatal screening for the CAH which is currently not a mandatory practice in some countries. Early diagnosis through screening at birth can contribute to better outcomes and prognosis.

## AUTHOR CONTRIBUTIONS

All individuals designated as authors and co‐authors have significantly contributed to this case report. Detailed contribution is as follows: Author 1—primary physician working with the patient, contributed in the section of case presentation. Author 2—Lead the team, contributed to the Abstract and Introduction parts. Author 3—Worked on the discussion section. Author 4‐ helped in review, editing, and made changes in the manuscript. Author 5‐ worked on reference format, reviewed the manuscript, and contributed to the submission process.

## CONFLICT OF INTEREST

The authors declare that they have no conflicts of interest.

## ETHICAL APPROVAL

This case report was approved by Suraksha Women and Children Hospital, Hyderabad, India.

## DISCLOSURE

The content of this publication is solely the responsibility of the authors and does not necessarily represent official views of the institutions the author belongs to.

## CONSENT

Written informed consent was obtained from the parents of the child in their vernacular language to publish this report in accordance with the journal's patient consent policy.

## Data Availability

The literature review data used to support the findings of this study are included within the article.
